# Deterministic Overlapping Multimorbidity Phenotypes for Leakage-Safe EHR Modeling of Incident Cognitive Impairment in All of Us

**DOI:** 10.3390/jdream6020009

**Published:** 2026-05-28

**Authors:** Zahra Rahemi, Meisam Omidi

**Affiliations:** 1 School of Nursing, Clemson University, Clemson, SC 29634, USA; 2 School of Dentistry, Marquette University, Milwaukee, WI 53233, USA

**Keywords:** multimorbidity phenotyping, deterministic rule functions, overlap-aware representation, leakage-safe modeling, association rule mining, k-modes clustering, electronic health records, cognitive impairment

## Abstract

**Background:**

In electronic health record (EHR) research, multimorbidity is commonly represented by summary indices that may oversimplify disease co-occurrence or by unsupervised cluster labels that may lack stability across samples. This study evaluated a leakage-safe framework for deterministic, overlapping multimorbidity phenotyping.

**Methods:**

Data from the All of Us Research Program were used to study 23,435 adults aged 50 years and older, anchored at their first SARS-CoV-2-positive test. Eleven pre-index Charlson component indicators were used as the binary baseline representation. Stable co-occurrence patterns were explored using association rule mining with bootstrap recurrence criteria. K-modes clustering was used as a discovery aid, while final phenotype membership was defined by deterministic rule functions.

**Results:**

The framework produced three overlapping phenotype flags and a transparent 8-state overlap structure. The analytic cohort included 1462 incident cognitive impairment cases. Two phenotype flags were associated with higher odds of incident cognitive impairment. However, adding the phenotype flags to baseline models yielded only marginal improvements in discrimination.

**Conclusions:**

The framework’s primary value lies in providing a reproducible, transparent, and overlap-aware representation of multimorbidity rather than in producing substantial predictive gains. This approach may support interpretable baseline phenotyping and leakage-safe modeling in real-world EHR studies.

## 1. Introduction

Existing approaches to representing co-occurring chronic conditions in electronic health records (EHRs) fall into two broad categories, each carrying fundamental limitations that constrain their utility in risk modeling [1,2]. Scalar summary indices, such as the Charlson Comorbidity Index [3], offer operational convenience and portability but reduce high-dimensional disease configurations to a single value. In doing so, they discard the co-occurrence structures and inter-disease dependencies that are often statistically and clinically meaningful [4]. Conversely, unsupervised methods—including clustering and association rule mining—can recover higher-order patterns, but their outputs remain sensitive to algorithmic hyperparameters and sample perturbation [5–7]. This sensitivity undermines stability, reproducibility, and transportability across datasets and institutions [8]. Together, these shortcomings motivate the development of multimorbidity representations that are simultaneously structure-preserving, reproducible, and portable.

While the representational problem concerns how multimorbidity is encoded, a second and equally consequential challenge involves the temporal window of measurement [9]. In EHR-based risk modeling, predictors constructed from information recorded after a designated index event introduce temporal leakage, yielding inflated and misleading performance estimates [10,11]. Rigorous study designs must therefore enforce a strict separation between the pre-index baseline period for predictor derivation and the post-index follow-up period for outcome ascertainment [12–15]. This requirement is particularly vital when outcomes are identified from diagnostic codes, as variations in coding intensity and follow-up duration can systematically confound performance evaluations [13,14].

These challenges are not merely theoretical; they are especially consequential in clinical domains where multimorbidity drives risk and outcome measurement is inherently imprecise. The representational and temporal challenges outlined above are particularly acute in studies of incident cognitive impairment (CI) [16–18]. In this context, baseline risk is shaped by the totality of multimorbidity rather than isolated conditions, and outcome ascertainment typically depends on routine clinical coding [19,20]. Prior evidence on post-index cognitive risk remains inconsistent, largely because baseline cognition and pre-existing disease burdens are often incompletely characterized [21–23]. Complex chronic disease configurations, involving vascular, pulmonary, renal, hepatic, and malignant conditions, may influence or even mask index-associated risk signals [22]. Yet, these configurations are poorly captured by single-score summaries and lack the stability required for transportability when derived from unsupervised labels.

Incident cognitive impairment is a clinically relevant and methodologically demanding outcome for evaluating multimorbidity representations in real-world data. In older adults, CI risk is shaped by baseline multimorbidity and vascular disease burden rather than by a single isolated condition, and multimorbidity has been associated with elevated dementia risk in large cohort studies [19,21,23]. At the same time, EHR-based CI ascertainment is vulnerable to diagnostic timing, variable healthcare contact, and incomplete capture of clinically measured cognition, which are broader challenges in code-based EHR phenotyping and longitudinal outcome definition [1,9,24]. These features make CI an appropriate outcome for evaluating whether a leakage-safe, overlap-aware phenotype representation can preserve clinically meaningful baseline comorbidity structure while supporting interpretable association and risk-modeling analyses [24–26].

To bridge this gap, we propose a leakage-safe framework for deterministic, overlapping multimorbidity phenotyping using pre-index EHR data. Our approach utilizes eleven binary Charlson component indicators as an “atomic feature space” to ensure a reproducible foundation. Within this space, we explore co-occurrence patterns via association rule mining with bootstrap recurrence criteria. Crucially, we employ k-modes clustering solely as a discovery aid rather than a final labeling mechanism; final phenotype membership is defined by explicit deterministic rule functions mapped over the binary component vector. This process yields overlapping phenotype flags with directly computable prevalence, dependence, and a transparent overlap-state structure. The objective of this study is not to introduce an entirely new predictor domain, but rather to evaluate whether a deterministic, overlap-aware phenotype layer provides a more transparent and stable representation of baseline multimorbidity than either compressed summary scores or unstable unsupervised labels. This study is therefore methodology-first: incident CI association and prediction are used as evaluation tasks to assess whether deterministic, overlap-aware phenotype flags provide a transparent and reproducible representation of baseline multimorbidity under leakage-safe conditions.

## 2. Materials and Methods

### 2.1. Study Design, Cohort Definition, and Outcome Window

This study used a retrospective real-world data design based on electronic health record data from the All of Us Research Program. The analytic timeline was anchored at each participant’s first recorded positive SARS-CoV-2 test, which served as the index date. This index event was selected because it is a discrete, routinely recorded clinical event that provides a clear temporal anchor for separating pre-index baseline comorbidity history from post-index outcome ascertainment. The purpose of using this index was methodological rather than infection-specific: it enabled evaluation of the deterministic phenotype framework under a defined baseline-to-follow-up structure. To preserve temporal validity and prevent information leakage, all predictor variables were defined strictly from data recorded before the index date, whereas outcome ascertainment was restricted to the post-index period.

Participants were eligible if they were aged 50 years or older at index and had sufficient pre-index electronic health record history to derive baseline comorbidity indicators. Individuals with evidence of cognitive impairment before index were excluded so that the outcome represented incident post-index cognitive impairment rather than prevalent disease. Participants with missing age, sex, or race information were also excluded from adjusted analyses.

**Figure 1.**
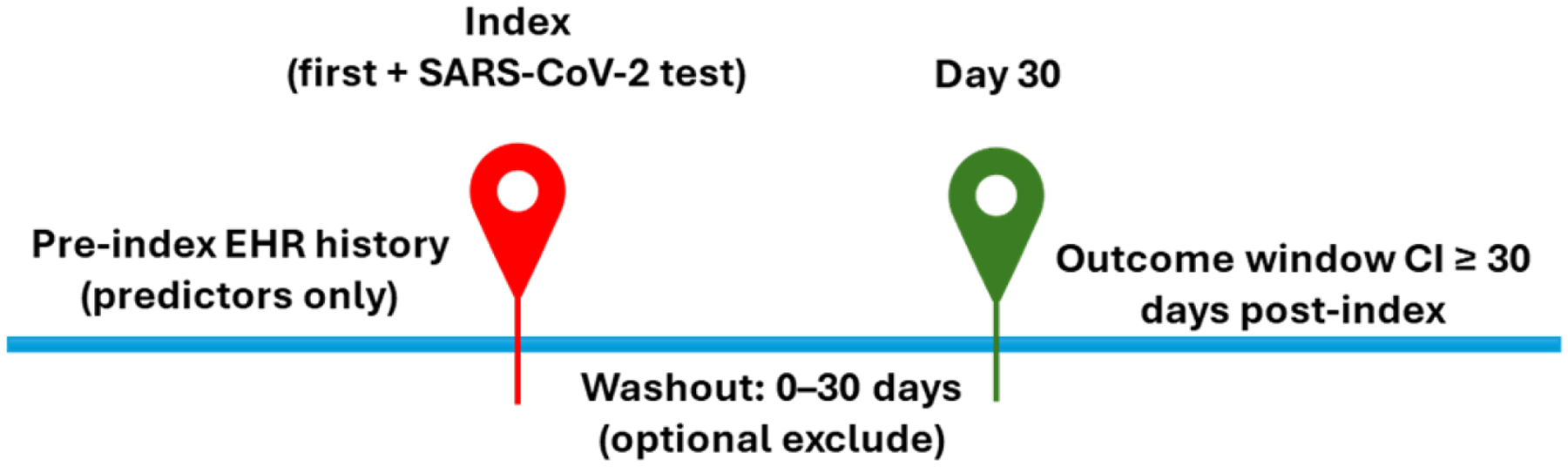
Study timeline and analytic windows. Schematic of study design. Baseline predictors were derived exclusively from pre-index EHR history. The index date was the first SARS-CoV-2 positive test. A 0–30 day washout period was applied, and incident cognitive impairment (CI) was ascertained from day 30 post-index through the last recorded EHR activity date.

The primary outcome was incident cognitive impairment identified from diagnosis codes recorded at least 30 days after the index date. This day-30 threshold was used to reduce the risk of counting acute or immediately concurrent coding events as incident outcomes and to maintain separation between baseline representation and post-index outcome assessment. Participants without a qualifying post-index cognitive impairment code were treated as non-cases in the primary analysis. This leakage-safe baseline-to-outcome design was used consistently for phenotype discovery, association analyses, and prediction modeling. Cohort assembly and exclusions are summarized in Figure 1.

### 2.2. Baseline Multimorbidity Representation

Baseline multimorbidity was represented using eleven binary Charlson component indicators derived exclusively from pre-index ICD-10-CM diagnosis codes [27]. Each indicator denoted the presence or absence of one Charlson comorbidity domain before the index date [3], so that each participant was represented by a binary vector *x* ∈ {0, 1}^11^. This binary component vector was treated as the atomic baseline representation because it preserves condition-level structure and permits explicit modeling of co-occurrence patterns, overlap, and threshold-based phenotype definitions.

In parallel, an age-adjusted Charlson comorbidity index score was computed as a conventional scalar summary measure for descriptive comparison [28]. However, the primary methodological focus of this study was not the scalar score itself, but the higher-dimensional binary representation from which deterministic overlapping phenotype flags could be constructed. Relative to a single summary index, the binary component vector retains multimorbidity structure that would otherwise be compressed, including partial nesting of conditions and heterogeneous combinations with the same total burden [25,29].

All baseline comorbidity variables were defined strictly from diagnoses recorded before the index date. No post-index diagnosis information was used in constructing baseline multimorbidity features, phenotype rules, association covariates, or prediction inputs.

### 2.3. Discovery of Stable Co-Occurrence Structure by Association Rule Mining

To explore recurrent multimorbidity structure in the binary baseline representation, we applied association rule mining (ARM) to the eleven Charlson component indicators. ARM was used as a discovery operator to identify stable and interpretable co-occurrence patterns, rather than as a final phenotype-labeling method. Rules were evaluated using standard association measures, including support, confidence, and lift, and only rules meeting prespecified minimum thresholds were retained for further consideration.

To reduce sensitivity to sampling variation, rule stability was assessed by bootstrap recurrence analysis. In each bootstrap resample, ARM was rerun using the same tuning thresholds, and each candidate rule was tracked across resamples. Rules were considered stability-supported if they recurred in at least 70% of bootstrap samples and remained clinically interpretable. This procedure was used to prioritize co-occurrence patterns that were both empirically recurrent and suitable for deterministic downstream encoding. ARM results were not used directly as participant-level labels. Instead, stability-supported rule patterns were carried forward as one input into the engineering of deterministic overlapping phenotype flags.

### 2.4. Discovery Support from K-Modes Clustering

K-modes clustering was applied to the eleven-dimensional binary baseline comorbidity vectors as a secondary discovery operator to summarize dominant multimorbidity structure. Because the input variables were binary categorical indicators, k-modes was preferred over Euclidean-based partitioning methods. Clustering was implemented using Cao initialization, Hamming (simple matching) dissimilarity, and multiple random restarts, with candidate solutions evaluated for *k* = 2 to 6.

Clustering was not used to define final participant-level phenotypes. Instead, it served as a diagnostic tool to examine whether recurrent co-occurrence structure in the binary baseline representation was compatible with a small number of interpretable multimorbidity patterns. Candidate solutions were assessed using within-cluster cost, minimum cluster size, and interpretability of the resulting cluster structure. Because cluster assignments are mutually exclusive and sensitive to modeling choices, the clustering step was used only to support pattern discovery and rule engineering. Final phenotype definitions were therefore specified deterministically from the original baseline component indicators rather than inherited directly from cluster labels.

### 2.5. Deterministic Engineering of Overlapping Phenotype Flags

Final participant-level phenotypes were defined deterministically from the eleven-dimensional baseline comorbidity vector rather than from unsupervised cluster labels. Let *x* ∈ {0, 1}^11^ denote the pre-index Charlson component vector for a participant. Three phenotype flags were defined as Boolean functions on *x*:

(1) $$f_{1}(x)=1(\text{malignancy}=1\wedge \text{chronic pulmonary disease}=1)$$

(2) $$f_{2}(x)=1(\text{cerebrovascular disease}=1\wedge \text{congestive heart failure}=1$$

(3) $$f_{3}(x)=1\left(\overset{11}{\underset{j=1}{\sum }}x_{j}\geq 3\right).$$

The induced phenotype map is therefore

(4) $$F(x)=(f_{1}(x),f_{2}(x),f_{3}(x))\in {\left\{0,\;1\right\}}^{3},$$

which defines an explicit 8-state overlap space.

This deterministic construction was chosen to preserve interpretability and reproducibility while allowing overlap between clinically meaningful multimorbidity patterns. Unlike mutually exclusive cluster labels, the phenotype flags can co-occur by design, making partial nesting and shared burden explicit rather than forcing participants into a single group. Because *f*_3_ is defined by a threshold on the total number of active Charlson components, some overlap states may be structurally impossible under the joint rule system. In particular, simultaneous positivity for the Cancer–COPD and Cerebrovascular–Heart Failure flags necessarily implies at least three active Charlson components, so the state (1, 1, 0) is structurally absent.

### 2.6. Association Modeling for Incident Cognitive Impairment

Associations between phenotype membership and incident cognitive impairment were estimated using multivariable logistic regression. The primary association model included the three phenotype flags jointly, together with age, sex, race, and baseline Charlson component count as adjustment covariates. Joint inclusion of the phenotype flags was used because the deterministic construction allows overlap by design, so separate one-flag models would not appropriately account for shared phenotype membership.

The adjusted odds ratio for each phenotype therefore represents the association between that phenotype flag and incident cognitive impairment conditional on the other phenotype flags and demographic covariates. Baseline Charlson component count was included as a parsimonious adjustment for overall multimorbidity burden while allowing the phenotype coefficients to reflect structured co-occurrence beyond simple disease accumulation. To evaluate whether the phenotype layer improved explanatory fit beyond demographics alone, we also compared a reference model containing age, sex, and race with an expanded model that additionally included the jointly entered phenotype flags using a likelihood-ratio test.

### 2.7. Baseline Risk Prediction Models

To evaluate the predictive contribution of the deterministic phenotype layer, we fit elastic-net logistic regression models for incident cognitive impairment using leakage-safe pre-index predictors only. Data were partitioned into a 70:30 training-test split with a fixed random seed (2026), and model tuning was performed in the training set using 5-fold cross-validation. Because incident cognitive impairment was less frequent than non-case status, inverse-frequency class weights were applied during model fitting.

Two baseline-risk representations were evaluated. The Baseline model used demographic variables together with the age-adjusted Charlson comorbidity index as a scalar summary of baseline burden. The Baseline + Phenotypes model added the three deterministic overlapping phenotype flags to the same baseline predictor set. This comparison was designed to test whether the phenotype layer provided incremental predictive value beyond a conventional scalar baseline comorbidity summary.

Model performance was assessed in the held-out test set using the area under the receiver operating characteristic curve, area under the precision-recall curve, Brier score, calibration intercept, calibration slope, and decision-curve analysis. Bootstrap confidence intervals for discrimination and calibration metrics were estimated from repeated resampling of the test set.

### 2.8. Subgroup Robustness and Sensitivity Analyses

Robustness of predictive performance was examined in prespecified subgroup analyses stratified by sex-at-birth and race group, restricted to strata with adequate event counts for stable estimation. In addition, exploratory continuous net reclassification improvement (NRI) was computed on the held-out test set to assess risk re-ranking between the two prediction models. These analyses were interpreted as supplementary and exploratory rather than as primary evidence of improved clinical utility.

Sensitivity analyses were also performed excluding participants with baseline metastatic cancer to assess robustness to this high-severity subgroup. Sensitivity analyses were conducted for both phenotype association models and prediction performance.

### 2.9. Software

All analyses were conducted using Python 3.10 within the secure All of Us Researcher Workbench environment.

## 3. Results

### 3.1. Cohort Assembly and Baseline Characteristics

A total of 23,435 participants met the eligibility criteria for the primary leakage-safe analytic cohort, including 1462 participants with incident cognitive impairment and 21,973 without incident cognitive impairment. Cohort assembly, exclusions, and the final analytic sample are summarized in Figure 1.

**Figure 2.**
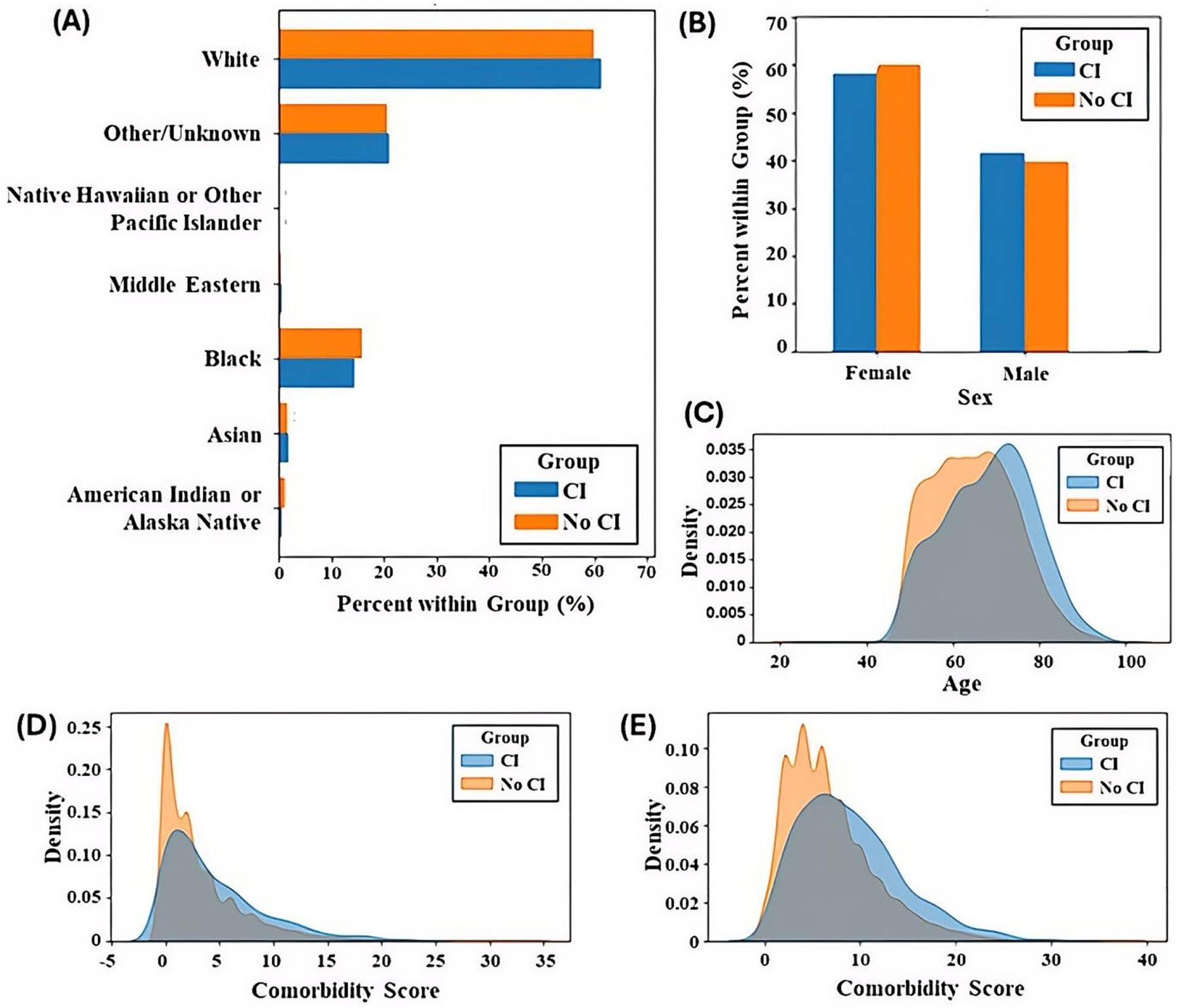
Race, sex, age, and baseline comorbidity burden in CI versus No-CI groups. (**A**) Race group distribution (normalized within group). (**B**) Sex-at-birth distribution (normalized within group). (**C**) Kernel density of age at index (first SARS-CoV-2 positive test). (**D**) Density of baseline (pre-index) Charlson comorbidity score. (**E**) Density of age-adjusted baseline Charlson score (residualized on age at index). All density curves are normalized within group.

Baseline characteristics of the analytic cohort are reported in Table 1. Participants with incident cognitive impairment were older than non-cases and had higher baseline comorbidity burden, including higher Charlson and age-adjusted Charlson scores. The incident cognitive impairment group also showed higher prevalences of several baseline Charlson component domains, particularly cerebrovascular disease, congestive heart failure, chronic pulmonary disease, peripheral vascular disease, and renal disease. Figure 2 visualizes between-group differences in race distribution (Figure 2A), sex-at-birth (Figure 2B), age at index (Figure 2C), baseline Charlson score (Figure 2D), and age-adjusted baseline Charlson score (Figure 2E). Detailed comparisons of individual baseline Charlson component prevalences between incident cognitive impairment cases and non-cases are provided in Supplementary Table S1.

### 3.2. Deterministic Phenotype Prevalence and Overlap Structure

In the discovery stage, association rule mining and k-modes clustering were used to examine recurrent structure in the binary baseline comorbidity space. The retained association rules and clustering diagnostics, summarized in Supplementary Tables S2 and S3, were not used directly as participant-level labels. Instead, these discovery-stage results informed the construction of the final deterministic overlapping phenotype map. Exact rule definitions, manuscript labels, and phenotype prevalences are provided in Supplementary Table S4. This design separated exploratory pattern detection from the reproducible rule-based phenotype representation used in the primary analyses.

The deterministic phenotype construction yielded three overlapping baseline multimorbidity flags with distinct prevalences in the analytic cohort. The High multimorbidity burden phenotype was the most common, present in 7120 participants (30.38%), followed by the Cancer–COPD phenotype in 3928 participants (16.76%) and the Cerebrovascular–Heart Failure phenotype in 1807 participants (7.71%) (Table 2).

Because phenotype membership was defined by deterministic Boolean rules rather than by mutually exclusive clustering, overlap between phenotype flags was directly observable and quantifiable. The full overlap structure is reported in Supplementary Tables S4 and S5. As expected from the rule construction, the overlap state (1,1,0) was structurally absent because simultaneous positivity for the Cancer–COPD and Cerebrovascular–Heart Failure phenotypes necessarily implies at least three active Charlson components and therefore positivity for the High multimorbidity burden phenotype.

**Figure 3.**
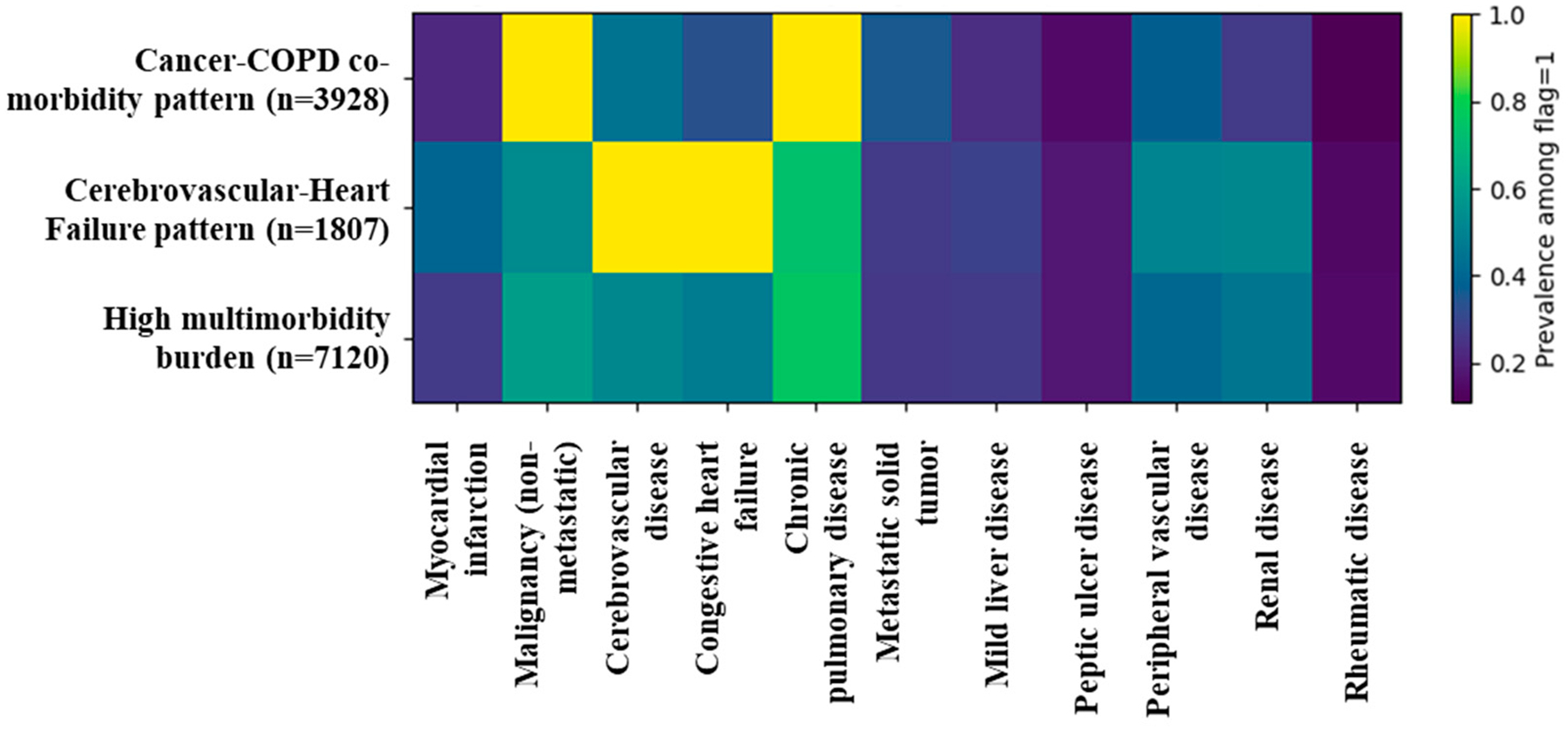
Phenotype fingerprints for overlapping multimorbidity phenotype flags. Heatmap showing prevalence of the 11 baseline Charlson component indicators among participants positive for each phenotype flag: Cancer–COPD co-morbidity pattern, Cerebrovascular–Heart Failure pattern, and High multimorbidity burden (≥3 Charlson components). Values represent within-flag prevalence.

Pairwise dependence between phenotype flags is summarized in Supplementary Table S5 and S6. These overlap and dependence patterns indicate that the phenotype layer captures structured co-occurrence and partial nesting within the baseline comorbidity space rather than imposing mutually exclusive participant labels. Figure 3 shows the component-level fingerprints of the three phenotypes across the eleven baseline Charlson domains.

### 3.3. Associations Between Phenotype Flags and Incident Cognitive Impairment

In multivariable logistic regression with the three phenotype flags entered jointly, the Cerebrovascular–Heart Failure phenotype and the High multimorbidity burden phenotype were each associated with higher odds of incident cognitive impairment after adjustment for age, sex, race, and baseline Charlson component count (Table 3). The adjusted odds ratio was 1.30 (95% CI 1.08–1.56; *p* = 0.005) for the Cerebrovascular–Heart Failure phenotype and 1.35 (95% CI 1.15–1.58; *p* = 0.0003) for the High multimorbidity burden phenotype. The Cancer–COPD phenotype was not significantly associated with incident cognitive impairment in the jointly adjusted model (OR 0.98, 95% CI 0.83–1.15; *p* = 0.80).

Because the phenotype flags were entered jointly, each coefficient reflects the association of that phenotype with incident cognitive impairment conditional on overlapping membership in the other deterministic phenotype flags and on the adjustment covariates. This joint specification is important because the phenotype layer was constructed to allow overlap rather than mutual exclusivity.

Overall, these results suggest that not all recurrent multimorbidity patterns contribute equally to baseline cognitive-impairment risk. In particular, the phenotype defined by concurrent cerebrovascular disease and heart failure, as well as the broader high-burden phenotype defined by at least three active Charlson components, showed stronger associations than the cancer–pulmonary pattern in this analytic setting.

### 3.4. Baseline Risk Prediction Performance

**Figure 4.**
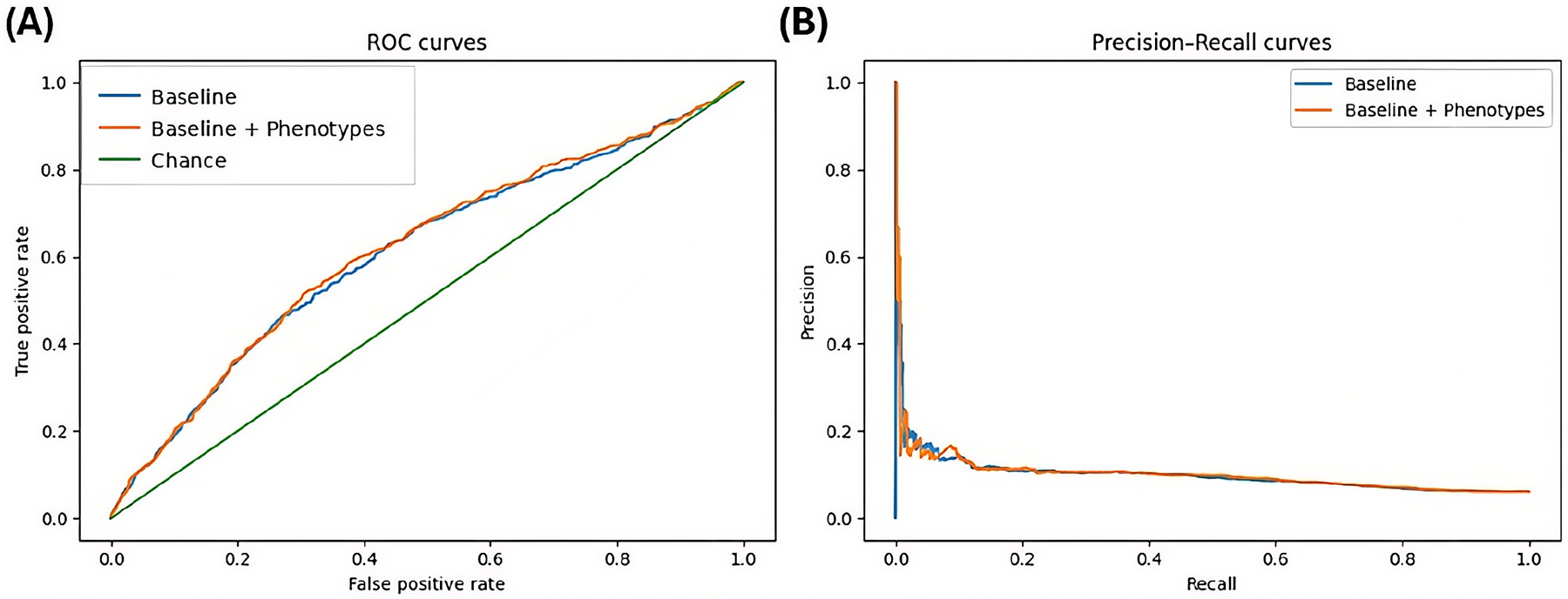
Discrimination performance for baseline risk prediction models. (**A**) Receiver operating characteristic (ROC) and (**B**) precision–recall (PR) curves on the held-out test set comparing the Baseline model (demographics + baseline Charlson score) and the Baseline + Phenotypes model (Baseline predictors plus the engineered phenotype flags). AUPRC is emphasized due to low outcome prevalence.

Baseline risk prediction performance is summarized in Table 4, and Figure 4 provides the corresponding visual comparison of the Baseline and Baseline + Phenotypes models. Relative to the baseline model, addition of the deterministic phenotype layer produced only modest changes in held-out predictive performance. AUROC increased from 0.612 to 0.618, and AUPRC increased from 0.100 to 0.101. The Brier score changed minimally, from 0.0551 to 0.0550, indicating little difference in overall probabilistic accuracy between the two models. Detailed predictive performance estimates with confidence intervals are provided in Supplementary Table S7. Calibration curves were similar between models (Figure S1), and exploratory decision curve analysis demonstrated comparable net benefit profiles across evaluated risk thresholds (Figure S2).

Calibration metrics were also similar across models. Calibration slope changed only slightly from 0.8395 in the Baseline model to 0.8414 after inclusion of phenotype flags, while the calibration intercept changed from −0.426 to −0.418. Taken together, these results indicate that the deterministic phenotype layer contributed little additional discrimination or calibration improvement beyond the baseline representation used in the primary prediction model.

These findings are consistent with the role of the phenotype layer as a structured and reproducible re-encoding of baseline multimorbidity information rather than as a major source of novel predictive signal. In this setting, the main value of the phenotype representation lies in interpretability, overlap-aware structure, and portability, while predictive gains remain limited.

### 3.5. Subgroup Robustness, Exploratory Reclassification, and Sensitivity Analyses

Subgroup predictive performance stratified by sex-at-birth and race group is reported in Table S8. Across these strata, the incremental gain from adding phenotype flags to the baseline model was small and heterogeneous.

Exploratory reclassification analysis suggested modest risk re-ranking. Continuous NRI was 0.196 (95% CI 0.105–0.288) comparing Baseline versus Baseline + Phenotypes on the held-out test set (Table S9). This result should be interpreted as evidence of limited re-ranking rather than of clinically meaningful utility improvement.

Sensitivity analyses excluding participants with baseline metastatic cancer are summarized in Tables S10 and S11. For association models, the overall pattern remained directionally similar, although precision decreased for some phenotype estimates after excluding this high-risk subgroup. For prediction models, removal of baseline metastatic cancer reduced precision-recall performance, consistent with lower outcome prevalence, while the difference between the two prediction models remained small.

## 4. Discussion

### 4.1. Principal Findings

This study evaluated a leakage-safe framework for representing baseline multimorbidity using deterministic overlapping phenotype flags derived from eleven pre-index Charlson component indicators. The primary contribution is methodological, whereas the incident CI association and prediction analyses serve as applied evaluations of the phenotype representation. In the All of Us case study anchored at first SARS-CoV-2 positivity, the proposed framework produced three interpretable phenotype flags with an explicit overlap structure, identified heterogeneous associations with incident cognitive impairment, and yielded only modest changes in predictive discrimination when added to the baseline model. The main contribution of the framework is therefore not a large increase in predictive performance, but a transparent and reproducible representation of multimorbidity structure that preserves overlap and partial nesting [30–32].

Two phenotype flags, the Cerebrovascular–Heart Failure phenotype and the High multimorbidity burden phenotype, were associated with higher odds of incident cognitive impairment in jointly adjusted models, whereas the Cancer–COPD phenotype was not [19,22,23,30]. This pattern suggests that deterministic co-occurrence structures defined on the same baseline component space are not interchangeable with respect to the outcome and may capture distinct aspects of multimorbidity burden beyond simple prevalence alone [11,26].

### 4.2. Methodological Contribution of Deterministic Overlapping Phenotypes

The central methodological contribution of this work is the construction of a deterministic phenotype layer that sits between a fully disaggregated binary comorbidity vector and a compressed scalar burden score. In many EHR studies, multimorbidity is represented either by a summary index, which is portable but structurally reductive, or by unsupervised labels, which may reveal co-occurrence structure but are often unstable under changes in initialization, algorithm selection, or sample composition [1,8,25]. The deterministic phenotype map evaluated here was designed to preserve clinically interpretable co-occurrence structure while remaining exactly reproducible once the baseline feature definitions are fixed [7,14,22].

A key advantage of this representation is that overlap is explicit rather than suppressed. Participants are not forced into mutually exclusive clusters, and partial nesting between phenotype flags can be quantified directly through the overlap cube and conditional dependence measures [33]. The structurally absent state (1,1,0) is particularly informative because it follows directly from the logical form of the phenotype rules, illustrating that the overlap structure is not arbitrary but internally constrained by the deterministic map itself [34,35]. This property is difficult to obtain from conventional clustering labels, which typically impose exclusivity even when the underlying disease patterns overlap [5–7].

The framework also supports transportability. Because final phenotype assignment depends only on a defined set of binary baseline indicators and fixed Boolean rules, the phenotype layer can in principle be reconstructed exactly across datasets that share comparable coding definitions [24]. This is a more stable basis for comparison than cohort-specific cluster labels whose interpretation may shift under routine analytic perturbations.

### 4.3. Interpretation of the Limited Predictive Gain

The phenotype layer yielded only small changes in held-out predictive performance. This result is not surprising and should not be interpreted as a failure of the framework [36,37]. The phenotype flags are deterministic functions of the same underlying baseline comorbidity components already available to the prediction model, so large improvements in discrimination would not be expected unless the phenotype layer captured interaction structure more efficiently than the original representation under the fitted model class. In this study, the observed changes in AUROC, AUPRC, and Brier score were minimal, indicating that the phenotype layer primarily reorganized existing baseline information rather than introducing a substantially new signal source [38,39].

This distinction is important. Representation can be valuable even when it adds little to conventional discrimination metrics. In the present setting, the phenotype layer improved interpretability by making structured co-occurrence and overlap explicit, supported association analyses that are easier to communicate than a high-dimensional component space alone, and provided a reproducible intermediate representation that may be useful for comparison across datasets and modeling settings. For this reason, the framework should be interpreted mainly as a representational and analytic tool rather than as a major predictive enhancement strategy [40].

From a practical standpoint, the deterministic phenotype layer is most useful when the goal is interpretable cohort stratification, reproducible subgroup definition, or comparison of multimorbidity structure across datasets. A scalar Charlson score remains appropriate when the goal is compact adjustment for overall comorbidity burden, but summary indices can obscure clinically relevant disease composition and may perform differently depending on index choice and data source [3,25,27,29]. Individual Charlson components may be preferable when the primary objective is prediction-focused modeling in sufficiently large datasets, because they preserve condition-level information; however, a full component representation can be less parsimonious and less easily communicated across clinical or epidemiologic audiences. The proposed phenotype flags occupy an intermediate position: they preserve clinically interpretable co-occurrence patterns, allow overlapping membership rather than forced assignment to a single mutually exclusive cluster, and can be reconstructed when the same baseline component definitions are available [8,24,33]. Thus, the framework may be most useful for epidemiologic and clinical informatics studies that require transparent multimorbidity profiling, subgroup reporting, transportability assessment, or robustness checks, rather than for direct clinical decision-making based on prediction alone.

### 4.4. Limitations, and Transportability

Several limitations should be considered. The outcome was defined from diagnosis codes recorded in routine EHR data rather than from standardized cognitive assessments, so misclassification of incident cognitive impairment is possible. Because participants differed in post-index healthcare contact and follow-up opportunity, the observed associations may also be influenced by surveillance bias and differential ascertainment. In addition, although the phenotype rules were designed to be transparent and stable, their final form still reflects analytic choices regarding thresholding, rule selection, and burden definition. The framework was evaluated in one real-world data setting anchored at SARS-CoV-2 positivity, and external validation in independent EHR environments is still needed. The use of first SARS-CoV-2 positivity as the index date should therefore be interpreted as a temporal anchoring strategy rather than as a claim that the phenotype framework is specific to COVID-19. The same design could be applied to other clinically meaningful index events, such as hospitalization, major surgery, acute cardiovascular events, or other infections, provided that baseline predictors can be defined before the index and outcomes are ascertained after an appropriate washout period. However, the empirical associations and predictive performance observed here may not generalize directly to non-COVID settings and should be externally evaluated before broader application.

These limitations do not negate the representational value of the approach, but they do constrain interpretation. The reported associations should not be treated as causal effects, and the modest predictive gains indicate that the phenotype layer is better suited to structured baseline characterization than to standalone risk discrimination. Future work could evaluate whether deterministic overlapping phenotype maps remain stable across other coding systems, disease domains, and temporal anchoring strategies, and whether they support downstream tasks such as subgroup discovery, calibration transfer, or structured interaction modeling more effectively than conventional summary scores or cohort-specific clusters.

## 5. Conclusions

In this real-world data case study, deterministic overlapping multimorbidity phenotype flags provided a transparent and reproducible representation of baseline comorbidity structure under a leakage-safe design. The resulting phenotype layer captured explicit overlap and partial nesting, supported interpretable association analyses, and showed only limited incremental predictive gain. These findings suggest that the main value of the framework lies in structured representation and portability rather than substantial improvement in conventional risk discrimination. Deterministic overlap-aware phenotype maps may be useful in other EHR applications where multimorbidity structure, reproducibility, and transportability are priorities.

## Supplementary Material

supplementary

The following supporting information can be downloaded at: https://www.mdpi.com/article/10.3390/jdream6020009/s1, Table S1: Baseline Charlson component prevalence comparisons between incident cognitive impairment cases and non-cases; Table S2: ICD-to-Charlson mapping used for baseline comorbidity feature construction; Table S3: Deterministic phenotype rule definitions, manuscript labels, and prevalences; Table S4: Overlap cube for the three deterministic phenotype flags; Table S5: Pairwise dependence measures and conditional overlap probabilities among phenotype flags; Table S6: Stability-supported association rules from association rule mining; Table S7: K-modes clustering diagnostics; Table S8: Detailed predictive performance metrics with confidence intervals; Table S9: Subgroup predictive performance by sex-at-birth and race group; Table S10: Exploratory continuous net reclassification improvement; Table S11: Sensitivity analysis for phenotype association models excluding participants with baseline metastatic cancer; Table S12: Sensitivity analysis for prediction models excluding participants with baseline metastatic cancer; Figure S1: Calibration curves for the Baseline and Baseline + Phenotypes models; Figure S2: Decision curve analysis for the Baseline and Baseline + Phenotypes models.

## Figures and Tables

**Table 1.** Baseline cohort characteristics by incident cognitive impairment status.

	Variable	CI	No-CI	SMD
**Age (years)**		68.3 (10.4)	64.7 (9.8)	0.363614
**Charlson score**		4.47 (4.51)	3.08 (3.70)	0.336420
**Sex At Birth**	Female	58.0%	60.0%	−0.040073
	Male	41.9%	40.0%	0.039319
**Race**	American Indian or Alaska Native	0.6%	1.4%	−0.074988
	Middle Eastern or North African	0.6%	0.4%	0.032006
	Asian	1.9%	1.8%	0.009132
	Black or African American	14.9%	16.0%	−0.030629
	White	60.7%	59.4%	0.026087
	Native Hawaiian or Other Pacific Islander	0.1%	0.1%	−0.008167
	None of these	21.2%	21.0%	0.006094
**Charlson comorbidities**	Myocardial infarction	13.3%	9.4%	0.123367
	Malignancy (any tumor)	37.2%	28.7%	0.180223
	Cerebrovascular disease	38.9%	20.4%	0.404764
	Congestive heart failure	24.6%	17.4%	0.176814
	Chronic pulmonary disease	55.3%	44.2%	0.222234
	Metastatic solid tumor	13.8%	9.1%	0.146683
	Mild liver disease	15.2%	11.2%	0.117072
	Peptic ulcer disease	10.3%	6.9%	0.119651
	Peripheral vascular disease	25.7%	14.8%	0.272410
	Renal disease	25.4%	19.3%	0.146537
	Rheumatic disease	10.0%	7.2%	0.099586

Notes: CI, incident cognitive impairment; No-CI, no incident cognitive impairment. Values are median (IQR) or n (%). Baseline comorbidities were computed from pre-index EHR history only. SMD, standardized mean difference. Total N = 23,435; CI n = 1462; No-CI n = 21,973.

**Table 2.** Prevalence and definitions of deterministic overlapping phenotype flags.

Phenotype Flag (Manuscript Label)	n (%)	Operational Definition (One Line)
Cancer–COPD co-morbidity pattern	3928 (16.76)	Baseline malignancy and chronic pulmonary disease both present.
Cerebrovascular–Heart Failure pattern	1807 (7.71)	Baseline cerebrovascular disease and congestive heart failure both present.
High multimorbidity burden (≥3 Charlson components)	7120 (30.38)	Baseline Charlson component count ≥ 3 (from 11 pre-index components).

Note: Phenotype flags were defined from 11 baseline Charlson component indicators. Exact Boolean rule definitions, code-variable mappings, overlap structure, and dependence metrics are provided in Supplementary Tables S3–S5. Exact deterministic rule definitions, code-variable mappings, overlap structure (8-state cube), and dependence metrics are reported in Supplementary Tables S4–S6.

**Table 3.** Adjusted associations between phenotype flags and incident cognitive impairment.

Phenotype Flag	Adjusted OR	95% CI	*p*-Value
Cancer–COPD co-morbidity pattern	0.98	0.83–1.15	0.80
Cerebrovascular–Heart Failure pattern	1.30	1.08–1.56	0.005
High multimorbidity burden (≥3 Charlson components)	1.35	1.15–1.58	0.0003

Note: OR = odds ratio; CI = confidence interval. The joint logistic regression model included all three phenotype flags and adjusted for age at index, sex at birth, race group, and baseline comorbidity burden. Associations are not interpreted causally.

**Table 4.** Predictive performance summary for baseline risk prediction models.

Model	AUC	AUPRC	Brier	Cal. Slope	Cal. Intercept
Baseline	0.612	0.100	0.0564	0.8395	−0.4261
Baseline + Phenotypes	*0.618*	*0.101*	*0.0564*	*0.8414*	*−0.4179*

Note: The Baseline model included demographics and baseline Charlson score. The Baseline + Phenotypes model added the three deterministic phenotype flags. AUC = area under the receiver operating characteristic curve; AUPRC = area under the precision–recall curve. Metrics were computed on the held-out test set.

## Data Availability

The data analyzed in this study were obtained from the All of Us Research Program and are subject to controlled-access and data use restrictions. Individual-level data cannot be publicly shared. Researchers who meet the criteria for access may request access through the All of Us Researcher Workbench. Derived analytic code, phenotype definitions, and summary outputs can be made available from the corresponding author upon reasonable request, subject to institutional and platform policies.

## References

[1] Voss RW; Schmidt TD; Weiskopf N; Marino M; Dorr DA; Huguet N; Warren N; Valenzuela S; O’Malley J; Quiñones AR Comparing Ascertainment of Chronic Condition Status with Problem Lists versus Encounter Diagnoses from Electronic Health Records. J. Am. Med. Inform. Assoc. JAMIA 2022, 29, 770–778. 35165743 10.1093/jamia/ocac016 PMC9006679

[2] Kharrazi H; Chi W; Chang HY; Richards TM; Gallagher JM; Knudson SM; Weiner JP Comparing Population-Based Risk-Stratification Model Performance Using Demographic, Diagnosis and Medication Data Extracted from Outpatient Electronic Health Records Versus Administrative Claims. Med. Care 2017, 55, 789–796. 28598890 10.1097/MLR.0000000000000754

[3] Charlson ME; Pompei P; Ales KL; MacKenzie CR A New Method of Classifying Prognostic Comorbidity in Longitudinal Studies: Development and Validation. J. Chronic Dis 1987, 40, 373–383. 3558716 10.1016/0021-9681(87)90171-8

[4] Stavem K; Hoel H; Skjaker SA; Haagensen R Charlson Comorbidity Index Derived from Chart Review or Administrative Data: Agreement and Prediction of Mortality in Intensive Care Patients. Clin. Epidemiol 2017, 9, 311. 28652813 10.2147/CLEP.S133624 PMC5476439

[5] Röchner P; Rothlauf F Unsupervised Anomaly Detection of Implausible Electronic Health Records: A Real-World Evaluation in Cancer Registries. BMC Med. Res. Methodol 2023, 23, 125. 37226114 10.1186/s12874-023-01946-0 PMC10207857

[6] Miotto R; Li L; Kidd BA; Dudley JT Deep Patient: An Unsupervised Representation to Predict the Future of Patients from the Electronic Health Records. Sci. Rep 2016, 6, 26094. 27185194 10.1038/srep26094 PMC4869115

[7] Mariam A; Javidi H; Zabor EC; Zhao R; Radivoyevitch T; Rotroff DM Unsupervised Clustering of Longitudinal Clinical Measurements in Electronic Health Records. PLoS Digit. Health 2024, 3, e0000628. 39405315 10.1371/journal.pdig.0000628 PMC11478862

[8] Lasko TA; Strobl EV; Stead WW Why Do Probabilistic Clinical Models Fail to Transport between Sites. npj Digit. Med 2024, 7, 53. 38429353 10.1038/s41746-024-01037-4 PMC10907678

[9] Beaney T; Clarke J; Woodcock T; Majeed A; Barahona M; Aylin P Effect of Timeframes to Define Long Term Conditions and Sociodemographic Factors on Prevalence of Multimorbidity Using Disease Code Frequency in Primary Care Electronic Health Records: Retrospective Study. BMJ Med. 2024, 3, e000474. 10.1136/bmjmed-2022-000474 PMC10868275 38361663

[10] Amanatidis A; Egan K; Nio K; Toma M Data-Leakage-Aware Preoperative Prediction of Postoperative Complications from Structured Data and Preoperative Clinical Notes. Surgeries 2025, 6, 87.

[11] Ferris JK; Wagar B; Choi A; Simkin J; Sbihi H; Harder K; Smolina K Temporal Multimorbidity Patterns and Cluster Identification: A Longitudinal Analysis of Administrative Data. BMC Med. 2025, 23, 370. 40597345 10.1186/s12916-025-04184-x PMC12219922

[12] Weeda ER; Kohn CG; Fermann GJ; Peacock WF; Tanner C; McGrath D; Crivera C; Schein JR; Coleman CI External Validation of Prognostic Rules for Early Post-Pulmonary Embolism Mortality: Assessment of a Claims-Based and Three Clinical-Based Approaches. Thromb. J 2016, 14, 7. 26977136 10.1186/s12959-016-0081-5 PMC4790043

[13] Kumar NK; Weeda ER; Wells PS; Peacock F; Fermann GJ; Wang L; Baser O; Schein J; Crivera C; Coleman CI; External Validation of a Clinical and Claims-Based Approach for Predicting 90-Day Post-Pulmonary Embolism Outcomes Among US Veterans. Blood 2016, 128, 533.

[14] Roy PM; Friou E; Germeau B; Douillet D; Kline JA; Righini M; Le Gal G; Moumneh T; Penaloza A Derivation and Validation of a 4-Level Clinical Pretest Probability Score for Suspected Pulmonary Embolism to Safely Decrease Imaging Testing. JAMA Cardiol. 2021, 6, 669–677. 33656522 10.1001/jamacardio.2021.0064 PMC7931139

[15] Newcomer JW; Ng-Mak D; Rajagopalan K; Loebel A Hospitalization Outcomes in Patients with Schizophrenia after Switching to Lurasidone or Quetiapine: A US Claims Database Analysis. BMC Health Serv. Res 2018, 18, 243. 29618351 10.1186/s12913-018-3020-2 PMC5885302

[16] Rahemi Z; Malatyali A; Adams SA; Jarrín OF; Demiris G; Parker V; Ghaiumy Anaraky R; Dye CJ Advance Care Planning Among Older Adults with Cognitive Impairment. Am. J. Hosp. Palliat. Med 2023, 40, 1182–1189. 10.1177/10499091221146255 PMC10282104 36541134

[17] Rahemi Z; Hashemi M; Omidi M Temporal Modeling of Cognitive Decline: Health Patterns and Disease Progression in the All of Us Dataset. Alzheimer’s Dement. 2025, 21, e108259.

[18] Rahemi Z; Hashemi M; Omidi M COVID-19 and Cognitive Decline: AI-Driven Insights into Risk Detection Among Older Adults Using All of Us Dataset. Alzheimer’s Dement. 2025, 21, e108255.

[19] Hu HY; Zhang YR; Aerqin Q; Ou YN; Wang ZT; Cheng W; Feng JF; Tan L; Yu JT Association between Multimorbidity Status and Incident Dementia: A Prospective Cohort Study of 245,483 Participants. Transl. Psychiatry 2022, 12, 505. 36476644 10.1038/s41398-022-02268-3 PMC9729184

[20] Zammit AR; Yu L; Oveisgharan S; Schneider JA; Bennett DA; Buchman AS Temporal Sequence of Incident Mild Cognitive Impairment, Incident Parkinsonism, and Risk of Death in Unimpaired Community-Dwelling Older Adults. J. Gerontol. Ser. A Biol. Sci. Med. Sci 2024, 80, glae275. 39545594 10.1093/gerona/glae275 PMC11701745

[21] Tsui A; Searle SD; Bowden H; Hoffmann K; Hornby J; Goslett A; Weston-Clarke M; Hamill Howes L; Street R; Perera R; The Effect of Baseline Cognition and Delirium on Long-Term Cognitive Impairment and Mortality: A Prospective Population-Based Study. Lancet Healthy Longev. 2022, 3, e232–e241. 35382093 10.1016/S2666-7568(22)00013-7 PMC7612581

[22] Berger M; Nadler JW; Browndyke J; Terrando N; Ponnusamy V; Cohen HJ; Whitson HE; Mathew JP Postoperative Cognitive Dysfunction: Minding the Gaps in Our Knowledge of a Common Postoperative Complication in the Elderly. Anesthesiol. Clin 2015, 33, 517. 26315636 10.1016/j.anclin.2015.05.008 PMC4555995

[23] El Husseini N; Katzan IL; Rost NS; Blake ML; Byun E; Pendlebury ST; Aparicio HJ; Marquine MJ; Gottesman RF; Smith EE Cognitive Impairment Following Ischemic and Hemorrhagic Stroke: A Scientific Statement from the American Heart Association/American Stroke Association. Stroke 2023, 54, e272–e291. 37125534 10.1161/STR.0000000000000430 PMC12723706

[24] Carrell DS; Floyd JS; Gruber S; Hazlehurst BL; Heagerty PJ; Nelson JC; Williamson BD; Ball R A General Framework for Developing Computable Clinical Phenotype Algorithms. J. Am. Med. Inform. Assoc. JAMIA 2024, 31, 1785–1796. 38748991 10.1093/jamia/ocae121 PMC11258420

[25] Stirland LE; González-Saavedra L; Mullin DS; Ritchie CW; Muniz-Terrera G; Russ TC Measuring Multimorbidity beyond Counting Diseases: Systematic Review of Community and Population Studies and Guide to Index Choice. BMJ 2020, 368, m160. 32071114 10.1136/bmj.m160 PMC7190061

[26] Coste J; Valderas JM; Carcaillon-Bentata L Estimating and Characterizing the Burden of Multimorbidity in the Community: A Comprehensive Multistep Analysis of Two Large Nationwide Representative Surveys in France. PLoS Med. 2021, 18, e1003584. 33901171 10.1371/journal.pmed.1003584 PMC8109815

[27] Quan H; Sundararajan V; Halfon P; Fong A; Burnand B; Luthi JC; Saunders LD; Beck CA; Feasby TE; Ghali WA Coding Algorithms for Defining Comorbidities in ICD-9-CM and ICD-10 Administrative Data. Med. Care 2005, 43, 1130–1139. 16224307 10.1097/01.mlr.0000182534.19832.83

[28] Goldstein LB; Samsa GP; Matchar DB; Horner RD Charlson Index Comorbidity Adjustment for Ischemic Stroke Outcome Studies. Stroke 2004, 35, 1941–1945. 15232123 10.1161/01.STR.0000135225.80898.1c

[29] Wei MY; Luster JE; Ratz D; Mukamal KJ; Langa KM Development, Validation, and Performance of a New Physical Functioning–Weighted Multimorbidity Index for Use in Administrative Data. J. Gen. Intern. Med 2021, 36, 2427–2433. 33469748 10.1007/s11606-020-06486-7 PMC8342661

[30] Maya Viejo JD; Navarro Ros FM Automated Chronic Obstructive Pulmonary Disease Phenotyping and Control Assessment in Primary Care: Retrospective Multicenter Study Using the Seleida Model. JMIR Med. Inform 2025, 13, e74932. 41082606 10.2196/74932 PMC12517459

[31] Bzdok D; Wolf G; Kopal J Harnessing Population Diversity: In Search of Tools of the Trade. GigaScience 2024, 13, giae068. 39331809 10.1093/gigascience/giae068 PMC11427908

[32] Desvarieux M; Rundek T; Ahsan H; Narvaez J; Diaz F; Malinsky D; Ruiz LM; Topaz M; Falconer T; Natarajan K; Accelerating Real-World Prediction and Research in Alzheimer’s: The M3AD Study. Alzheimer’s Dement. 2026, 22, e71174. 41848005 10.1002/alz.71174 PMC13093339

[33] Agarwal N; Ahmed HA; Bhattacharyya DK Non-Exclusive Clustering: A Partitioning Approach. In Proceedings of the 2015 2nd International Conference on Emerging Information Technology and Engineering Solutions, Mahashtra, India, 20–21 February 2015; pp. 7–12.

[34] Brun-Usan M; de Juan García J; Latorre R A Minimal CA-Based Model Capturing Evolutionarily Relevant Features of Biological Development. Mathematics 2025, 13, 3238.

[35] Munsamy G; Ayres G; Greco C; Kam K; Minto-Cowcher G; St. John J; Bohnuud T; Bakalar M; Chow W; Pecoraro R; Designing AI-Programmable Therapeutics with the EDEN Family of Foundation Models. bioRxiv 2026.

[36] Cooper DN; Krawczak M; Polychronakos C; Tyler-Smith C; Kehrer-Sawatzki H Where Genotype Is Not Predictive of Phenotype: Towards an Understanding of the Molecular Basis of Reduced Penetrance in Human Inherited Disease. Hum. Genet 2013, 132, 1077–1130. 23820649 10.1007/s00439-013-1331-2 PMC3778950

[37] Lailvaux SP; Mishra A; Pun P; Kabir MWU; Wilson RS; Herrel A; Hoque MT Machine Learning Accurately Predicts the Multivariate Performance Phenotype from Morphology in Lizards. PLoS ONE 2022, 17, e0261613. 35061733 10.1371/journal.pone.0261613 PMC8782310

[38] Schinkel M; Boerman AW; Paranjape K; Wiersinga WJ; Nanayakkara PWB Detecting Changes in the Performance of a Clinical Machine Learning Tool over Time. eBioMedicine 2023, 97, 104823. 37793210 10.1016/j.ebiom.2023.104823 PMC10550508

[39] Beaumont JL; Lin HM; Goodman E; Yu H; Geiger A; Hudgens S Establishing Meaningful Change Thresholds in PatientReported Outcomes Among Patients with Anaplastic Lymphoma Kinase-Positive Non–Small Cell Lung Cancer in ALTA-1L Trial. Value Health 2024, 27, 182–189. 37951539 10.1016/j.jval.2023.10.014

[40] Tonekaboni S; Friedman SF; Zhang X; Maddah M; Uhler C A Representation Fusion Framework for Decoupling Diagnostic Information in Multimodal Learning. npj Digit. Med 2025, 8, 765. 41408105 10.1038/s41746-025-02144-6 PMC12712021

