# Supplementary material for: Deterministic Overlapping Multimorbidity Phenotypes for Leakage-Safe EHR Modeling of Incident Cognitive Impairment in All of Us
Source: J Interdiscip Res Appl Med. Author manuscript; Available in PMC 2026 Jun 12. (PMC13256376; doi:10.3390/jdream6020009)
Supplement: supplementary [file NIHMS2182166-supplement-supplementary.pdf]

Supplementary

Deterministic Overlapping Multimorbidity Phenotypes for Leakage-Safe EHR Modeling of Incident Cognitive Impairment in All of Us

Zahra Rahemi [1] and Meisam Omidi [2,*]

[1] School of Nursing, Clemson University, Clemson, SC 29634, USA
[2] School of Dentistry, Marquette University, Milwaukee, WI 53233, USA
* Correspondence: meisam.omidi@marquette.edu

Supplementary Methods

ICD-to-Charlson Mapping

Pre-index Charlson comorbidity indicators were derived by mapping ICD-9-CM and ICD-10-CM diagnosis codes recorded before the index date to Charlson component categories using a validated coding framework based on Quan et al. Each Charlson domain was converted to a binary indicator denoting whether at least one qualifying diagnosis code was present before index. The complete code lists and component mappings used in the analytic pipeline are provided in Table S12.

All Charlson component indicators used for phenotype construction, association modeling, and prediction were computed strictly from pre-index diagnosis records to preserve a leakage-safe baseline design. Post-index diagnosis information was not used to define any baseline multimorbidity feature in the primary analysis.

Follow-up Definition and Outcome Window

The index date was defined as the first recorded positive SARS-CoV-2 PCR or antigen test in the electronic health record. Incident cognitive impairment ascertainment began on day 30 after index to preserve temporal separation between baseline covariate construction and post-index outcome assessment. End of follow-up was defined as the last recorded EHR activity date available for each participant. Participants without documented EHR follow-up beyond 30 days after index were not considered at risk for the primary outcome window and were excluded from the primary analytic cohort.

Outcome Definition (Incident CI)

Incident cognitive impairment was defined by the first new ICD-9-CM or ICD-10-CM diagnosis code corresponding to mild cognitive impairment or dementia recorded at least 30 days after the index date. Participants with any documented evidence of mild cognitive impairment or dementia before the index date were excluded so that the outcome represented incident post-index cognitive impairment rather than prevalent disease.

Association Rule Mining (ARM): Thresholds and Bootstrap Stability

Association rule mining was applied to the eleven binary Charlson component indicators to identify recurrent co-occurrence patterns in baseline multimorbidity structure. ARM was used as a discovery operator rather than as a final participant-labeling method. Candidate rules were evaluated using standard association metrics, including support, confidence, and lift. In the primary discovery analysis, rules were screened using prespecified minimum thresholds of support, confidence, and lift, and only clinically interpretable rules satisfying all thresholds were retained for stability assessment.

To reduce sensitivity to sampling variation, rule stability was evaluated by bootstrap recurrence analysis using 5500 bootstrap resamples. In each bootstrap resample, ARM was rerun using the same tuning thresholds, and each candidate rule was tracked across resamples. Rules were considered stability-supported if they recurred in at least 70% of bootstrap samples and remained clinically interpretable. Stable retained rules are summarized in Table S2 and were carried forward as one input into deterministic phenotype construction.

K-Modes Clustering: Discovery Settings and Stability Diagnostics

K-modes clustering was applied to the eleven-dimensional binary baseline comorbidity vectors as a secondary discovery operator to summarize dominant multimorbidity structure. Because the input variables were binary categorical indicators, k-modes was preferred over Euclidean-based partitioning methods. Clustering was implemented using Cao initialization, Hamming (simple matching) dissimilarity, and multiple random restarts, with candidate solutions evaluated over a prespecified range of cluster numbers.

Clustering was not used to define final participant-level phenotypes. Instead, it served as a diagnostic tool to evaluate whether recurrent co-occurrence structure in the binary baseline representation was compatible with a small number of interpretable multimorbidity patterns. Candidate solutions were assessed using within-cluster cost, minimum cluster size, and interpretability of the resulting cluster structure. The selected solution was interpreted jointly with the ARM output and descriptive prevalence structure to support deterministic phenotype construction. Discovery clustering diagnostics are summarized in Table S3.

Deterministic Phenotype Flag Implementation

Three phenotype flags were implemented deterministically from the pre-index Charlson component indicators used in the analytic pipeline. Internal coding variables were as follows:

- Cancer-COPD co-morbidity pattern: canc_pre = 1 AND copd_pre = 1
- Cerebrovascular-Heart Failure pattern: cevd_pre = 1 AND chf_pre = 1
- High multimorbidity burden (≥3 Charlson components): charlson_component_count ≥ 3, where charlson_component_count equals the sum of the 11 pre-index Charlson component indicators.

Table S3 reports the exact deterministic rule definitions, manuscript labels, and phenotype prevalences. Table S4 reports the full 8-state overlap cube, including the structurally absent state (1,1,0). Table S5 reports pairwise dependence measures and conditional overlap probabilities.

Predictive Modeling Details

To evaluate the predictive contribution of alternative baseline multimorbidity representations, elastic-net logistic regression models were fit for incident cognitive impairment using leakage-safe pre-index

predictors only. Data were partitioned into training and test sets using a fixed random seed, and model tuning was performed in the training set using cross-validation. Because incident cognitive impairment was less frequent than non-case status, inverse-frequency class weights were applied during model fitting.

Predictive performance was assessed in the held-out test set using the area under the receiver operating characteristic curve, area under the precision-recall curve, Brier score, calibration intercept, calibration slope, and decision-curve analysis. Bootstrap confidence intervals for discrimination and calibration metrics were estimated from repeated resampling of the test set. Extended predictive performance summaries are reported in Tables S7-S9.

Table S1. Baseline covariate definitions and descriptive statistics.

Variable Name	*Type*	*Test Used*	*p-value*	*p-adjusted*	*Significant FDR*
Age	Continuous	Mann–Whitney U	2.2e-38	-	True
Sex At Birth	Categorical	Chi-Square	2.81e-01	3.28e-01	False
Race Group	Categorical	Chi-Square	1.26e-01	1.61e-01	False
AIDS/HIV (pre)	Categorical	Chi-Square	1.22e-01	1.59e-01	False
Myocardial Infarction (pre)	Categorical	Chi-Square	2.41e-06	4.32e-06	True
Any Malig-cy (pre)	Categorical	Chi-Square	2.99e-11	6.99e-11	True
Cerebrovascular Disease (pre)	Categorical	Chi-Square	3.96e-58	5.08e-57	True
Congestive Heart Failure (pre)	Categorical	Chi-Square	2.66e-11	6.41e-11	True
Chronic Pulmonary Disease (pre)	Categorical	Chi-Square	1.34e-15	4.71e-15	True
Dementia (pre)	Categorical	Chi-Square	2.68e-92	6.88e-91	True
Diabetes without Complications (pre)	Categorical	Chi-Square	7.72e-02	1.02e-01	False
Diabetes with Complications (pre)	Categorical	Chi-Square	3.07e-13	8.74e-13	True
Hemiplegia/Paraplegia (pre)	Categorical	Chi-Square	6.86e-09	1.43e-08	True
Metastatic Solid Tumor (pre)	Categorical	Chi-Square	1.26e-08	2.55e-08	True
Mild Liver Disease (pre)	Categorical	Chi-Square	7.14e-06	1.19e-05	True
Moderate-Severe Liver Disease (pre)	Categorical	Chi-Square	2.12e-02	2.87e-02	True
Peptic Ulcer Disease (pre)	Categorical	Chi-Square	3.12e-06	5.46e-06	True
Peripheral Vascular Disease (pre)	Categorical	Chi-Square	2.09e-27	1.24e-26	True
Renal Disease (pre)	Categorical	Chi-Square	3.93e-08	7.57e-08	True
Rheumatic Disease (pre)	Categorical	Chi-Square	1.05e-04	1.68e-04	True
Comorbidity Score Pre	Continuous	Mann–Whitney U	9.8e-36	-	True
Age Adjusted Comorbidity Score Pre	Continuous	Mann–Whitney U	3.6e-57	-	True

Table S2. Top association rules from ARM. Top baseline co-occurrence rules identified by ARM (support 0.02, confidence 0.15, lift 1.5, 5500 resamples; retention threshold ≥70% recurrence).

Rule ID	*Antecedent*	*Consequent*	*Support*	*Support (n)*	*Confidence*	*Lift*	*Bootstrap Stability*
R1	Cancer and Peripheral Vascular Disease	Metastatic Cancer	0.038660	906	0.471875	4.976773	1.000
R2	Metastatic Cancer	Cancer and Peripheral Vascular Disease	0.038660	906	0.407741	4.976773	1.000
R3	Cancer and Mild Liver Disease	Metastatic Cancer	0.025176	590	0.461298	4.865219	1.000
R4	Metastatic Cancer	Cancer and Mild Liver Disease	0.025176	590	0.265527	4.865219	1.000
R314	Acute Myocardial Infarction and Cancer	Metastatic Cancer	0.021208	497	0.450181	4.747973	0.992
R315	Metastatic Cancer	Acute Myocardial Infarction and Cancer	0.021208	497	0.223672	4.747973	0.992
R5	Metastatic Cancer	Cancer and Cerebrovascular Disease	0.044165	1035	0.465797	4.615621	1.000
R6	Cancer and Cerebrovascular Disease	Metastatic Cancer	0.044165	1035	0.437632	4.615621	1.000
R7	Congestive Heart Failure and Peripheral	Acute Myocardial Infarction	0.026755	627	0.415782	4.264273	1.000

	Vascular Disease						
R8	Acute Myocardial Infarction	Congestive Heart Failure and Peripheral Vascular Disease	0.026755	627	0.274398	4.264273	1.000
R9	Cancer and Congestive Heart Failure	Metastatic Cancer	0.030041	704	0.402977	4.250115	1.000
R10	Metastatic Cancer	Cancer and Congestive Heart Failure	0.030041	704	0.316832	4.250115	1.000
R11	Cerebrovascular Disease and Congestive Heart Failure	Acute Myocardial Infarction	0.030851	723	0.400111	4.103542	1.000
R12	Acute Myocardial Infarction	Cerebrovascular Disease and Congestive Heart Failure	0.030851	723	0.316411	4.103542	1.000
R13	Acute Myocardial Infarction and Renal Disease	Congestive Heart Failure	0.030211	708	0.732919	4.071098	1.000
R14	Congestive Heart Failure	Acute Myocardial Infarction and Renal Disease	0.030211	708	0.167812	4.071098	1.000
R15	Cerebrovascular Disease and Metastatic Cancer	Peripheral Vascular Disease	0.027438	643	0.592081	3.796284	1.000
R16	Peripheral Vascular Disease	Cerebrovascular Disease and Metastatic Cancer	0.027438	643	0.175923	3.796284	1.000

R17	Metastatic Cancer	Cancer and Chronic Pulmonary Disease	0.060209	1411	0.635014	3.788580	1.000
R18	Cancer and Chronic Pulmonary Disease	Metastatic Cancer	0.060209	1411	0.359216	3.788580	1.000
R19	Cerebrovascular Disease and Peripheral Vascular Disease	Metastatic Cancer	0.027438	643	0.356430	3.759199	1.000
R20	Metastatic Cancer	Cerebrovascular Disease and Peripheral Vascular Disease	0.027438	643	0.289379	3.759199	1.000
R21	Acute Myocardial Infarction and Peripheral Vascular Disease	Congestive Heart Failure	0.026755	627	0.674194	3.744898	1.000
R22	Congestive Heart Failure and Renal Disease	Acute Myocardial Infarction	0.030211	708	0.364948	3.742918	1.000
R23	Acute Myocardial Infarction	Congestive Heart Failure and Renal Disease	0.030211	708	0.309847	3.742918	1.000
R24	Cancer and Congestive Heart Failure	Acute Myocardial Infarction	0.026413	619	0.354322	3.633929	1.000
R25	Acute Myocardial Infarction	Cancer and Congestive Heart Failure	0.026413	619	0.270897	3.633929	1.000

R26	Acute Myocardial Infarction and Cerebrovascular Disease	Congestive Heart Failure	0.030851	723	0.643238	3.572954	1.000
R27	Congestive Heart Failure	Acute Myocardial Infarction and Cerebrovascular Disease	0.030851	723	0.171368	3.572954	1.000
R28	Acute Myocardial Infarction and Cerebrovascular Disease	Peripheral Vascular Disease	0.026541	622	0.553381	3.548147	1.000
R29	Peripheral Vascular Disease	Acute Myocardial Infarction and Cerebrovascular Disease	0.026541	622	0.170178	3.548147	1.000
R30	Peripheral Vascular Disease	Acute Myocardial Infarction and Cancer	0.025987	609	0.166621	3.536925	1.000
R31	Acute Myocardial Infarction and Cancer	Peripheral Vascular Disease	0.025987	609	0.551630	3.536925	1.000
R32	Cerebrovascular Disease and Peripheral Vascular Disease	Acute Myocardial Infarction	0.026541	622	0.344789	3.536166	1.000
R33	Acute Myocardial Infarction	Cerebrovascular Disease and Peripheral Vascular Disease	0.026541	622	0.272210	3.536166	1.000

R34	Acute Myocardial Infarction	Congestive Heart Failure and Chronic Pulmonary Disease	0.040495	949	0.415317	3.516243	1.000
R35	Congestive Heart Failure and Chronic Pulmonary Disease	Acute Myocardial Infarction	0.040495	949	0.342847	3.516243	1.000
R36	Metastatic Cancer	Chronic Pulmonary Disease and Peripheral Vascular Disease	0.033326	781	0.351485	3.419284	1.000
R37	Chronic Pulmonary Disease and Peripheral Vascular Disease	Metastatic Cancer	0.033326	781	0.324201	3.419284	1.000
R38	Acute Myocardial Infarction and Chronic Pulmonary Disease	Congestive Heart Failure	0.040495	949	0.614239	3.411875	1.000

Table S3. k-modes discovery diagnostics. Discovery clustering diagnostics for k-modes applied to the 11 baseline Charlson components (k = 2–6; n_init = 20; Hamming/simple matching distance).

k	*n_init*	*distance*	*cost*	*min_cluster_%*
2	20	Hamming (matching dissimilarity)	35138.0	0.4506
3	20	Hamming (matching dissimilarity)	30364.0	0.0743
4	20	Hamming (matching dissimilarity)	28170.0	0.0929
5	20	Hamming (matching dissimilarity)	25768.0	0.1268
6	20	Hamming (matching dissimilarity)	26206.0	0.0519

Table S4. Deterministic phenotype flag definitions and prevalence. Exact deterministic rule definitions for each phenotype flag, code-variable mappings, and prevalence (n, %) in the final analytic cohort (N = 23,435).

Phenotype label (manuscript)	***Deterministic rule (pre-index only)***	***n***	***%***
Cancer-COPD co-morbidity pattern	(Malignancy = 1) AND (Chronic pulmonary disease = 1)	3,928	16.76
Cerebrovascular-Heart Failure pattern	(Cerebrovascular disease = 1) AND (Congestive heart failure = 1)	1,807	7.71
High multimorbidity burden (≥3 Charlson components)	Charlson component count ≥ 3	7,120	30.38

Table S5. Overlap structure (8-state cube) for phenotype flags. Counts and proportions for the full 8-state overlap cube across the three phenotype flags (Cancer–COPD, Cerebrovascular–Heart Failure, High multimorbidity). The structurally absent state (Cancer–COPD = 1, Cerebrovascular–Heart Failure = 1, High multimorbidity = 0) is reported explicitly.

State	*Cancer-COPD*	*Cerebrovascular-HF*	*High multimorbidity*	*n*	*%*
000	0	0	0	15,613	66.62
001	0	0	1	2,879	12.29
010	0	1	0	53	0.23
011	0	1	1	962	4.11
100	1	0	0	649	2.77
101	1	0	1	2,487	10.61
110	1	1	0	0	0.00
111	1	1	1	792	3.38

Note: State 110 is structurally absent under these deterministic definitions because Cancer-COPD implies malignancy and Cerebrovascular-HF implies cerebrovascular disease and heart failure; these conditions contribute to the Charlson component count used to define High multimorbidity (≥3).

Table S6. Dependence and conditional overlap between phenotype flags. Pairwise dependence between phenotype flags summarized by phi coefficients and conditional overlap probabilities to quantify nesting and overlap structure.

Pair of phenotype flags	***Phi (φ)***	***Conditional overlap (most informative direction)***
Cancer-COPD vs High multimorbidity	0.518	P(High multimorbidity = 1 | Cancer-COPD = 1) = 3,279/3,928 = **83.48%**
Cerebrovascular-HF vs High multimorbidity	0.419	P(High multimorbidity = 1 | Cerebrovascular-HF = 1) = 1,754/1,807 = **97.07%**
Cancer-COPD vs Cerebrovascular-HF	0.209	P(Cerebrovascular-HF = 1 | Cancer-COPD = 1) = 792/3,928 = **20.17%**

Table S7. Detailed predictive performance metrics with bootstrap confidence intervals.

Model	***AUC (95% CI)***	***AUPRC (95% CI)***	***Brier (95% CI)***	***Cal. Slope (95% CI)***	***Cal. Intercept (95% CI)***
Baseline	0.612 (0.582–0.644)	0.100 (0.084–0.121)	0.0564 (0.051–0.061)	0.8395 (0.7975–0.8815)	-0.426 (-0.531 – -0.321)
Baseline + Phenotypes	0.618 (0.588–0.648)	0.101 (0.086–0.121)	0.0564 (0.051–0.061)	0.8414 (0.7574–0.9255)	-0.418 (-0.520 – -0.312)

Table S8. Subgroup predictive performance by sex-at-birth and race group.

level	*n*	*events*	*AUC (Baseline)*	*AUC (Baseline + Phenotypes)*	*ΔAUC*
Sex					
Female	4194	252	0.574360	0.583451	0.009091
Male	2836	174	0.663983	0.665841	0.001858
Race					
Black or African American	1144	69	0.670307	0.673576	0.003269
White	4194	258	0.576663	0.589057	0.012394

Table S9. Exploratory reclassification results (continuous NRI). Exploratory continuous net reclassification improvement (NRI) comparing Baseline versus Baseline + Phenotypes predictions on the held-out test set, with bootstrap confidence intervals. Results are interpreted as re-ranking measures and not as evidence of clinical utility improvement.

Comparison	***Reclassification metric***	***Estimate***	***95% CI***	***Notes***
Baseline** vs **Baseline** + **Phenotypes	Continuous NRI (total)	0.196	0.105–0.288	Computed on the held-out test set; bootstrap CI.

Table S10. Sensitivity analyses excluding baseline metastatic cancer. Phenotype associations with incident CI (joint adjusted model)

Phenotype flag	***OR***	***95% CI***	***p-value***
Panel A. Primary cohort (includes metastatic cancer)			
Cancer–COPD co-morbidity pattern	0.979	0.828–1.157	0.802
Cerebrovascular–Heart Failure pattern	1.295	1.080–1.554	0.0053
High multimorbidity burden (≥3 components)	1.353	1.149–1.593	0.00029
Panel B. Sensitivity cohort (excludes metastatic cancer)			
Cancer–COPD co-morbidity pattern	0.926	0.766–1.119	0.424
Cerebrovascular–Heart Failure pattern	1.129	0.906–1.406	0.279
High multimorbidity burden (≥3 components)	1.299	1.078–1.565	0.0059

Note: Joint models include all three phenotype flags and adjust for age at index, sex at birth, race group, and baseline comorbidity burden. Associations are not interpreted causally.

Table S11. Prediction performance sensitivity excluding metastatic cancer

Evaluation set	*Model*	*AUC*	*AUPRC*	*Brier*
Full test set	Baseline	0.612	0.100	0.0564
Full test set	Baseline + Phenotypes	0.618	0.101	0.0563
Test set excluding metastatic cancer	Baseline	0.609	0.0909	0.0557
Test set excluding metastatic cancer	Baseline + Phenotypes	0.615	0.0953	0.0556

Note: Metrics were computed using existing held-out test predictions from the prediction cohort (n=22,794; train=15,955; test=6,839; outcome prevalence ≈0.062). The lower AUPRC after excluding metastatic cancer reflects removal of a higher-risk subgroup.

Table S12. ICD-9 and ICD-10 Code Lists for Charlson Categories

Charlson Category	***ICD-9 Codes***	***ICD-10 Codes***
Myocardial Infarction *(MI)*	410.x, 412.x	I21.x, I22.x, I25.2
Congestive Heart Failure *(CHF)*	428.x, 402.01, 402.11, 402.91, 404.01, 404.03, 404.11, 404.13, 404.91, 404.93	I50.x, I11.0, I13.0, I13.2
Peripheral Vascular Disease *(PVD)*	440.2x, 443.9x, 441.x, 785.4, V43.4	I70.x, I73.9, I71.x, I72.x, I74.x, I77.x, I79.x, I80.x, I83.x, I87.0x, I87.1x, I87.2x, I87.9x
Cerebrovascular Disease *(CVD)*	430.x–434.x, 436.x, V12.54	I60.x–I64.x, I69.x, G45.x
Dementia	290.x	F01.x, F02.x, F03.x, G30.x
Chronic Pulmonary Disease *(CPD)*	490.x–496.x, 500.x–505.x	J40.x–J47.x, J60.x–J67.x, J68.4x, J70.1x–J70.3x
Rheumatologic Disease *(Connective Tissue)*	710.x–714.x	M05.x–M06.x, M32.x, M33.x, M34.x, M35.3x, M36.x
Peptic Ulcer Disease *(PUD)*	531.x–534.x	K25.x–K28.x
Mild Liver Disease	571.2x, 571.4x–571.5x, 571.6x–571.8x	B18.x, K70.0x–K70.3x, K71.x, K72.0x–K72.2x, K73.x, K74.x, K76.0x, K76.2x–K76.4x, K76.8x–K76.9x
Diabetes without Complication *(DM w/o)*	250.0x–250.3x	E10.0x–E10.3x, E11.0x–E11.3x, E12.0x–E12.3x, E13.0x–E13.3x, E14.0x–E14.3x
Diabetes with Complication *(DM w/ comp.)*	250.4x–250.9x	E10.4x–E10.9x, E11.4x–E11.9x, E12.4x–E12.9x, E13.4x–E13.9x, E14.4x–E14.9x
Hemiplegia / Paraplegia	342.x, 344.0x–344.1x	G81.x, G82.x
Renal Disease	585.x, 586.x, V42.0, V56.0, V56.1	N18.x, N19.x, Z99.2x
Any Malignancy (excluding Skin)	140.x–172.x, 174.x–195.x, 200.x–208.x	C00.x–C26.x, C30.x–C34.x, C37.x–C41.x, C43.x–C58.x, C60.x–C76.x, C81.x–C85.x, C88.x, C90.x–C96.x
Moderate/Severe Liver Disease	456.0x–456.2x, 572.2x–572.4x	K72.1x–K72.9x, K76.6x, B18.2x
Metastatic Solid Tumor	196.x–199.x	C77.x–C80.x
AIDS/HIV	042.x	B20.x–B24.x

ICD-9 = International Classification of Diseases, Ninth Revision, Clinical Modification
ICD-10 = International Classification of Diseases, Tenth Revision, Clinical Modification
CCI = Charlson Comorbidity Index

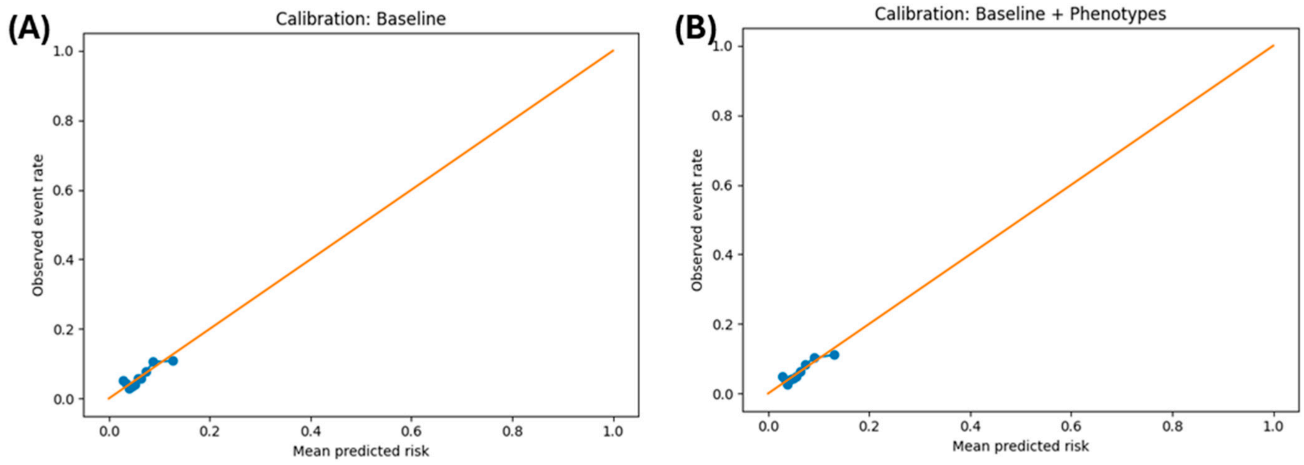

Figure S1. Calibration curves for baseline risk prediction models. Calibration curves on the held-out test set comparing the (A) Baseline and (B) Baseline + Phenotypes models. The diagonal line indicates perfect calibration.

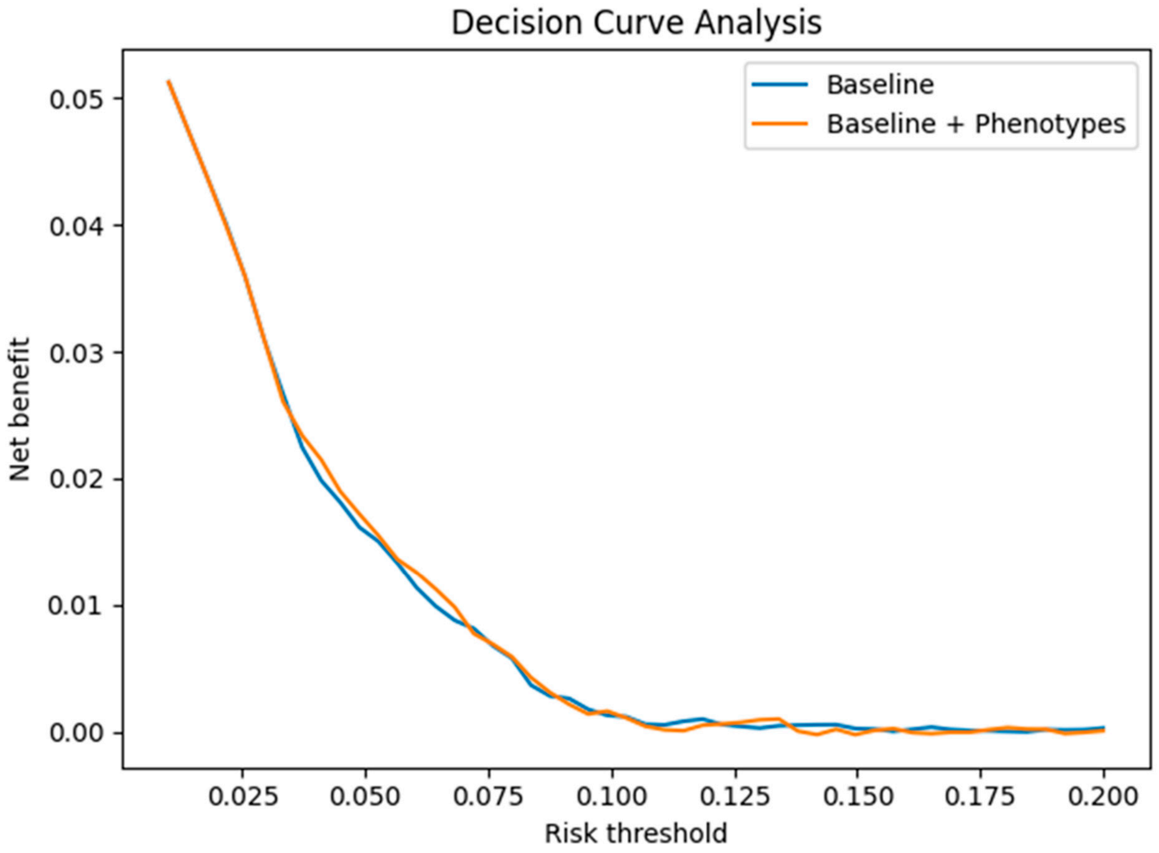

Figure S2. Decision curve analysis for baseline risk prediction models. Decision curve analysis on the held-out test set comparing net benefit across risk thresholds for the Baseline and Baseline + Phenotypes models. "Treat-all" and "treat-none" strategies are shown as reference curves.
